# Gold Nanoparticles Synthesis Using Stainless Steel as Solid Reductant: A Critical Overview

**DOI:** 10.3390/nano10040622

**Published:** 2020-03-27

**Authors:** Margherita Izzi, Maria C. Sportelli, Luciana Tursellino, Gerardo Palazzo, Rosaria A. Picca, Nicola Cioffi, Ángela I. López Lorente

**Affiliations:** 1 Department of Chemistry, University of Bari “Aldo Moro”, Via Orabona, 4, 70126 Bari, Italy; margherita.izzi@uniba.it (M.I.); maria.sportelli@uniba.it (M.C.S.); lucianakturs@gmail.com (L.T.); gerardo.palazzo@uniba.it (G.P.); 2 Departamento de Química Analítica, Instituto Universitario de Investigación en Química Fina y Nanoquímica IUIQFN, Universidad de Córdoba, Campus de Rabanales, Edificio Marie Curie, E-14071 Córdoba, Spain; q32loloa@uco.es

**Keywords:** gold nanoparticle, stainless steel corrosion, plasmonic particle, aqueous metal colloid

## Abstract

Gold nanoparticles (AuNPs) were produced using stainless steel as a solid reductant to assist the synthesis of metal NPs, using HAuCl_4_ as a precursor. This method is very easy, quick, and cost-effective, allowing the synthesis of highly stable NPs without additional capping agents. However, the reaction mechanism is still under debate. In order to contribute to the investigation of the synthesis of AuNPs using stainless steel, different experimental conditions were tested. Cl^−^ concentration, pH of the precursor solution, as well as stainless steel composition were systematically changed. The syntheses were performed recording the open circuit potential to potentiometrically explore the electrochemical properties of the system, under *operando* conditions. Spectroscopic and morphological characterizations were carried out along with potentiometric monitoring, aiming at correlating the synthesis parameters with the AuNPs characteristics. As a result, an overview of the process features, and of its most reasonable mechanism were obtained.

## 1. Introduction

Gold nanoparticles (AuNPs) are the most ancient metal nanophase ever used and they are the most common metal nanomaterial employed even today. This success is justified by the broad number of synthetic procedures available, which allows a fine tailoring of size, shape, and surface properties, providing a wide range of applications. In addition, the (relatively) high chemical and physical stability, the ease of surface functionalization with organic or biological functionalities, and the multitude of plasmon-related optical properties has led to an indisputable predominance in nanoscience [1,2,3,4,5,6]. Nowadays, their employment is predominantly focused in the biomedicine field for therapeutic [7,8], diagnostic [9], imaging [10], or drug delivery applications [11], as well as in the analytical field for sensing and biosensing applications [1,12,13,14,15,16].

Starting from Turkevich [17] and Brust–Schiffrin [18] classical methods to synthesize AuNPs, several and various routes have been implemented and examined, commonly classified in chemical, physical, electrochemical and biological synthetic methods. Chemical colloidal synthesis is one of the most commonly used method to synthesize AuNPs [19] and involves: (i) a metal precursor, such as gold (III) derivatives (i.e. HAuCl_4_), (ii) a reductant, and (iii) a stabilizer [20,21,22]. In some cases, the excess reductant can also behave as stabilizer. Chemical synthesis of AuNPs is a low-cost and easily scalable technology, which provides quite reproducible results (in terms of size and shape). However, some wet chemical processes suffer for drawbacks related to the use of toxic solvents, contamination from chemical precursors and production of potential hazardous by-products [20]. To overcome these disadvantages, other approaches are becoming popular in nanomaterial applications. Among them, laser ablation synthesis in solution is a synthetic route classified as a physical method which could be a satisfactory alternative. It ensures a good biocompatibility (especially if it is carried out in aqueous environment) because it allows producing colloids of a relatively high purity, without any potential toxic chemical and by-product. However, it incurs high investment costs, and the most diffused laser sources are not capable of producing nanomaterials on an industrial scale [23]. Colloid electrochemical synthesis is based on the sacrificial anode electrolysis [24]. This method provides colloidal solutions without a chemical reductant and contamination by its sub-products [25]. At the same time, it requires a specialized setup and peculiar experimental conditions (e.g. a conductive electrolyte, specific potential values, etc). Alternative biological green synthesis methods, which use microorganisms, plant extracts, intracellular or extracellular extracts of fungi or bacteria as potential biofactories for the synthesis of AuNPs, have been introduced and have become a new trend in nanoparticle production [19,26], offering nontoxicity and a reproducible production [27]. However, biosynthesis approaches to the production of metal nanoparticles require specific laboratory conditions and settings [28]. Furthermore, a careful purification of synthesized nanoparticles from impurities, such as the microbes themselves, is necessary [29]. 

An innovative method to produce AuNPs was suggested by Han’s [30] and López-Lorente’s [31] research groups, which allows the synthesis of AuNPs from tetrachloroauric acid solution using steel or stainless steel as solid reducing agent. This method is extremely simple, one-pot, low-cost, non-toxic and eco-friendly [32]. In fact, it avoids the use of a reductant in solution and can be carried out under mild conditions, at atmospheric pressure and room temperature. Moreover, it is an excellent candidate for potentially large-scale AuNPs production [31]. This synthesis is based on the reduction of Au^3+^ to Au^0^ by means of a stainless steel piece which is simultaneously oxidized. The mechanism leading to the formation of AuNPs remains elusive. Some reaction pathways have been proposed. The first hypothesis assumes that when the stainless steel is immersed in an aqueous solution of HAuCl_4_·3H_2_O, Cl^−^ ions released from the Au precursor are transported through the oxide film (commonly composed by oxides of Fe, Cr and Ni) to the metal surface, resulting in the corrosion of the stainless steel. The consequent release of electrons is used for the reduction of Au^3+^ to Au^0^ [30,31]. According to the second hypothesis, the reaction mainly occurs in the bulk of the solution, when the hydrogen formed from the stainless steel assisted reduction of protons in the precursor acidic media reduces AuCl_4_^−^ ions, yielding AuNPs [31]. In order to contribute to the investigation of this system, we explored its electrochemical aspects. The AuNP syntheses were performed monitoring the open circuit potential (OCP), to explore the electrochemical behaviour of steel and track the solution potential. The *operando* OCP monitoring has never been examined and it is useful to assess the role of steel corrosion in the synthesis mechanism. Furthermore, to deepen the abovementioned theories on the synthesis mechanism, the OCP tracking was also examined under different Cl^−^ concentration and pH values; in this way, their role on steel corrosion (correlating to AuNP formation mechanism) was assessed. Additionally, the reaction time, the precursor solution concentration, as well as the composition of the steel passivation layer were also investigated. It was found that steel composition was extremely important in tuning the NP synthesis yield and colloid properties (i.e. size, zeta potential, stability). Iron seems to play the main role, though chromium and nickel are responsible for the modification of the corrosion behaviour. The proper tuning of these components may be used to tailor gold nanoparticles.

## 2. Materials and Methods 

### 2.1. Materials

In the proposed syntheses, gold(III) chloride trihydrate (HAuCl_4_·3H_2_O, 99.9+%, Sigma-Aldrich, Milan, Italy) was used as a gold precursor. Stainless steel rods (GoodFellow Cambridge Ltd, Huntingdon, UK, length 10 cm, diameter 1 mm, total area 3.2 cm^2^) were used as reductant. Three stainless steels of different composition were used, labelled according to AISI (American Iron and Steel Institute) classification:

- AISI 430 (Fe = 81%, Cr = 17%, Mn, Si, C, S, P as minor components)
- AISI 410 (Fe = 87.5%, Cr = 12.5%)
- AISI 304 (Fe = 72%, Cr = 18%, Ni = 10%)

Sulfuric acid (H_2_SO_4_, 95-98%, ACS reagent, Sigma-Aldrich, Milan, Italy) and sodium hydroxide (NaOH, 98.0%, ACS reagent, Sigma-Aldrich) were used to adjust pH. Sodium chloride (NaCl, Sigma-Aldrich, Milan, Italy) was employed to investigate [Cl^−^] effect. Aqueous solutions were prepared in Milli-Q water (25°C, 18.2 MΩ). Aqua regia was prepared as a 1:3 mixture of nitric acid (HNO_3_, 67%, TraceSelect^®^, Sigma-Aldrich, Milan, Italy) and hydrochloric acid (HCl, 37%, TraceSelect^®^, Fluka Analytical Sigma-Aldrich, Milan, Italy). Transmission electron microscopy (TEM) samples were prepared on Formvar^®^-coated, 300-mesh, Cu grids purchased from Agar Scientific (Stansted, Essex, UK). Scanning electron microscopy (SEM) and X-ray photoelectron spectroscopy (XPS) samples were prepared on Si slides from Si-Mat – Silicon Materials (Kaufering, Germany).

### 2.2. AuNPs Synthesis Using Stainless Steel Wire

Gold nanoparticles were synthesized according to the previously reported procedure [30,31], with some variations. The glassware was cleaned and sonicated for 5 minutes in freshly prepared aqua regia, followed by the same procedure using Milli-Q water. The stainless-steel substrates were subjected to a surface pre-treatment and cleaning procedure, consisting in polishing with sandpaper for about five minutes. Stainless steel rods shaped as spirals were employed. This form was useful to increase the active surface area and improve stir movement. As a general procedure, the reaction was started by immersing the stainless-steel piece under stirring into 15 mL of 1.3 mM HAuCl_4_·3H_2_O, aqueous solution. The reaction was stopped by extracting the steel from the solution. The proposed reaction was carried out at room temperature and atmospheric pressure. Freshly polished pristine steel rods were employed for AuNP production. Most of the syntheses were performed at pH = 3.5, acidifying the reaction medium with H_2_SO_4_, if necessary, before performing the synthesis in order to homogenize the starting pH conditions. All AuNPs syntheses were carried out while simultaneously recording OCP values. The OCP measurements were performed on a potentiostat model CHI 1140b (CH Instruments, Austin, TX, USA). The experimental setup, presented in Supplementary Figure S1, consisted of a two-electrode cell, including the stainless steel working electrode mounted on a rotating bar and an Ag/AgCl (KCl sat.) reference electrode. PAR model 636 (Oak Ridge, TN, USA) electrode rotator was operated at a constant rotation speed of 200 rpm throughout the experiments. The colloids synthesized using the three different stainless steels were labelled “430-AuNPs”, “410-AuNPs”, “304-AuNPs” respectively for AISI 430, AISI 410, AISI 304 steel. The [Cl^−^] and pH influence was studied using AISI 430. The effect of different Cl^−^ concentration (0.1 M, 0.5 M and 1 M) was studied adding NaCl. The effect of pH was studied at pH = 1, and 5. The lower pH value was obtained preparing the HAuCl_4_ solution in H_2_SO_4_ 0.05 M. Further, pH 5 was obtained adding a few µL of NaOH 1 M to the synthesis solution. 

### 2.3. Gold Colloids Characterization

UV-Vis spectra were acquired with a double beam spectrophotometer (Shimadzu UV-1601) in the 250−800 nm wavelength range. Quartz cuvettes (optical path 1 cm, Optech, München, Germany) were used. Localized surface plasmon resonance (LSPR) peak positions were estimated on at least three replicates for each type of nanocolloid. TEM was performed with a FEI Tecnai 12 microscope (Eindhoven, Netherlands), equipped with a LaB_6_ filament operating at 120 kV. Size distribution histograms were obtained with OriginPRO 2016 software, after TEM images processing performed by ImageJ software [33], manually highlighting individual NPs on each micrograph. Histograms were produced on three replicates, counting more than 800 nanoparticles. X-ray photoelectron spectroscopy measurements were performed on both steel rods and gold colloids deposited on silicon substrates, using a PHI Versaprobe II (Chanhassen, MN, USA) spectrometer equipped with monochromatized Al-Kα radiation (1486.6 eV), following a previously reported procedure [34,35]. Binding energy (BE) scale was corrected on C1s component at 284.8 eV. Au4f region was fitted using CasaXPS^®^ version 2.3.19PR1.0, selecting a sum function of Gaussian with a Lorentzian (SLG) for the Au^(0)^ component and a product function of a Gaussian with a Lorentzian (GL) for the Au^(I)^ and Au^(III)^ components.

## 3. Results and Discussion

Since the mechanism of this innovative approach is based on the steel corrosion, the electrochemical corrosion behaviour of steel was examined monitoring the OCP under *operando* conditions. First, the influence of stainless steel composition was examined. Second, the reaction time was evaluated. Finally, the chloride concentration and pH effects were explored. All the experiments were carried out using a HAuCl_4_ concentration of 1.3 mM, following previous studies by other groups [32].

### 3.1. Effect of the Stainless Steel Composition

**Figure 1:**
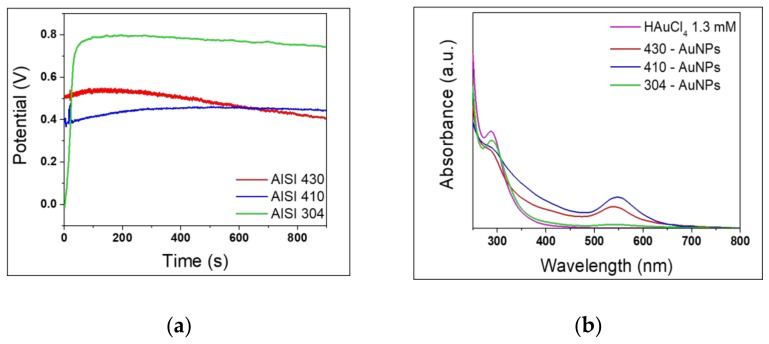
(**a**) OCP evolution during AuNP synthesis with different stainless steels: AISI 430, AISI 410 and AISI 304. (**b**) UV-Vis absorption spectra of the as-prepared AuNPs after 15 min of synthesis compared with the gold precursor UV-Vis spectrum.

In order to investigate the influence of the stainless-steel composition on the synthesis, AuNPs were produced using different steels, namely AISI 430, 410, and 304. In particular, they differ from each other in the chromium and nickel percentages. The syntheses were carried out for 15 minutes. Figure 1a shows the time-evolution of OCP for the aforementioned samples.

Considering the nature of the different stainless-steel samples, the initial potential values were substantially different. Especially, AISI 304 showed lower potentials compared to the other two, suggesting that it could be the more prone to corrosion [36], at least when the reaction starts. However, after about one minute, the AISI 304 potential reached more positive values then those of other steels, remaining then almost constant over all time. This means that the initial regime when corrosion is promoted is quickly replaced by a second one when the same process is limited. On the other hand, at the beginning AISI 430 and AISI 410 expressed more positive potentials than AISI 304, but OCP remained almost constant over time, or even slightly decreased. In particular, AISI 430 presented a decrement of about 100 mV over time. For AISI 410 and 430 stainless steel rods, the less positive OCP values and their trends upon reaction time suggest a higher trend to oxidation and a progressive dissolution process, which supported the AuNPs synthesis. Indeed, the corrosion and dissolution of steel metals such as iron, chromium and nickel, promote the reduction of Au^3+^ to Au^0^, as Han et al., and López Lorente et al., proposed [30,31]. The initial increment of OCP values frequently observed in the first minutes of reaction can be attributed to the formation of a metal oxide passive film on steel surface, involving iron, chromium and nickel species [37]. As the reaction proceeds, Cl^−^ ions are able to break the passive film and induce the formation of pits on the steel surface [38]. This mechanism would explain the progressive decrement of potentials, associated to the promotion of corrosion. The different corrosion behaviour is a result of the different steel composition. AISI 304 contains high chromium and nickel percentages. Both metals are known for forming passivation films. In particular, the nickel oxide passive film can be constantly broken and reformed, thus inhibiting the overall corrosion [39]. In this sense, AISI 304 shows the best corrosion resistance. By contrast, AISI 430, which has a Cr content comparable to that of AISI 304, contains manganese and sulphur traces; which tend to form manganese sulphides and manganese oxysulphides in the metal matrix, which turn out as preferential areas of corrosion initiation, deteriorating the corrosion resistance [40]. This could explain the progressive decrease of OCP values, which is not observed for AISI 410 containing only Cr.

Figure 1b presents the typical UV-Vis spectra of the as-synthesized nanocolloids using the three different steels together with the spectrum of the gold precursor solution. UV-Vis spectra were recorded in the range of 250−800 nm, unlike previous works which did not report the 250−400 nm range [30,31]. An absorption band at about 310 nm is observed for the 1.3 mM HAuCl_4_, which is in agreement with the yellowish colour of the gold precursor solution [41]. LSPR bands are evident in the UV-Vis spectra of the colloids, and their absorbance values are compatible with the OCP trends. In fact, 304-AuNPs obtained after 15 min display the lower LSPR absorbance at about 538 nm and, thus, the palest colour, while 430-AuNPs and 410-AuNPs UV-Vis spectra show more intense LSPR bands. Within the timescale of the reported syntheses, the low AuNP production exhibited by AISI 304 at room temperature is also in agreement with previous findings on the evolution of the stainless steel assisted synthesis of AuNPs reported in [31]. As explained before, AISI 430 and AISI 410 steels display an improved inclination to corrosion, which is strongly linked to NP production. Besides the LSPR maxima, all the spectra of colloids show a broad band in the 280−450 nm range. Different species can contribute to the absorption in this region. First, absorption around 300 nm can be ascribed to residual precursor, whose consumption occurs during the synthesis. In particular, a decrease in the intensity is generally observed in comparison to the absorption of the precursor solution. This behaviour is more pronounced for 410- and 430-AuNPs samples than for 304-AuNPs, in agreement with the different corrosion susceptibility of the three steels and, as a consequence, with the different AuNP production. On the other hand, after Fe^3+^ and Cr^3+^ ion release from steel surface, corresponding hydroxides and chloride mixed complexes are reasonably expected to be formed in solution, which leads to the increase in the baseline in the region 280−450 nm. Increasing chloride concentration deriving from gold precursor consumption can lead to the formation of various transition metal complexes absorbing in this region. In particular, at pH = 3.5 the most abundant complexes are FeOH^2+^, Fe(OH)_2_^+^ and CrOH^2+^, Cr_3_(OH)_4_^5+^ [42,43,44]. Consequently, this additional contribution cannot be separated from that of the gold precursor.

These evidences were corroborated by XPS analyses performed on both steel surface and gold colloids. Mechanically polished steel surfaces (before use) and their homologous samples used for AuNP synthesis (after use) were characterized. Relative atomic percentages of main elements found on steel surface together with Cr/Fe, Ni/Fe and Au/Fe ratios, before and after synthesis, are reported in Table 1 (overall compositions are reported in Table S1). A mere comparison of these data with the composition declared by the producer is not easy. XPS is a surface-sensitive technique providing information on the outer few nm of the sample’s surface. Considering the fact that surface composition can be extremely different from the bulk one, a direct comparison between the elemental compositions determined by different techniques and with different sampling depths cannot be done. Moreover, XPS analyses were performed on mechanically polished rods without preliminary Argon ion sputtering. For example, elements such as Mg, Ca, can be ascribed to residuals of the mechanical polishing. Carbon and organic oxygen are partially due to typical contamination observed in the analysis chamber.

On the other hand, XPS is typically applied to analyze steel surface, evidencing the formation of passive films as well as segregation [45,46]. The steel surface is mainly composed of oxide species of passivation film; hence, differences may exist between surface elemental percentages and theoretical bulk composition. In this view, we decided to report and use elemental percentage ratios more than the absolute %, being more informative of steel surface modification after use. Therefore, Cr/Fe, Ni/Fe and Au/Fe ratios are particularly useful to investigate corrosion effects on steel surface composition as well as the nature of gold colloids. Cr/Fe and Ni/Fe ratios can be compared with the theoretical values expected for the steels used in this study.

**Figure 2:**
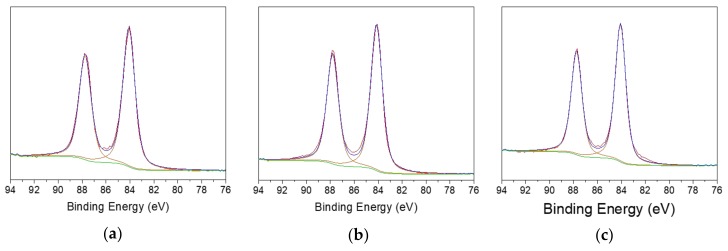
Au4f high resolution regions relevant to used (**a**) AISI 430, (**b**) AISI 410, (**c**) AISI 304.

Cr/Fe theoretical ratios are 0.2, 0.1, and 0.3 for AISI 430, 410, and 304, respectively. For example, it can be observed that Cr/Fe ratio for AISI 410 and AISI 304 keeps constant even after their use, indicating that both Fe and Cr are similarly dissolved. On the other hand, AISI 430 shows a decrease in Cr/Fe ratio after use, suggesting that chromium species segregate at steel surface. Cr/Fe ratios are slightly higher than nominal values, except for not used AISI 430. In this case, a certain contribution associated to the chromium segregated layer as well as the polishing of steel surface could be involved [47]. Considering the Ni/Fe ratio, its theoretical ratio for AISI 304 is 0.1 and the same value was found on polished surface. After use, Ni was barely detectable. According to these data, it can be inferred that the iron ion release in the solution, hence the steel corrosion, is more well-functioning for the AISI 430. After use, steel rods present a high amount of surface gold. Quite likely, gold metallization of steel surface is promoted over formation of colloidal AuNPs. The lowest Au% was observed for AISI 304 (Table 1), as expected by corrosion behaviour. Interestingly, the highest values were found for 410 and 430 steels. Therefore, the XPS evidences are in agreement with OCP results. Regarding Au speciation, Au4f region was acquired for all the three samples. Typical Au4f high resolution spectra are reported in Figure 2. For each steel sample, only one component, ascribed to Au(0) [48], at BE = 84.1 ± 0.2 eV was identified. This result agrees with the hypothesis that metallic gold was deposited on the steel surface during the synthesis. 

The relative atomic percentages of the main elements detected on the surface of dried colloids are reported in Table 2, together with the Cr/Fe, Au/Fe, and Ni/Fe ratios.

**Figure 3:**
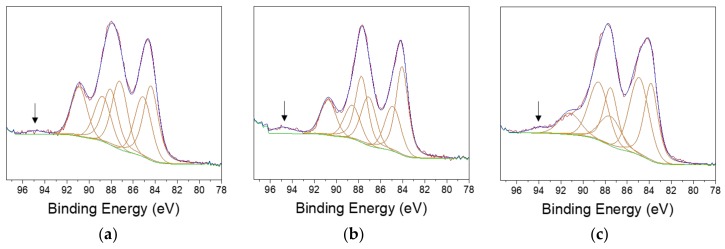
Au4f regions relevant to (**a**) 430-AuNPs, (**b**) 410-AuNPs, (**c**) 304-AuNPs. Arrows indicate the Fe3s component.

As can be observed, the gold percentage is higher in the 430-AuNP colloid; this suggests that the use of 430 steel promotes AuNP production. However, it can be noted that the Au/Fe ratios do not change with the different steel composition. Additionally, the colloid obtained by using AISI 430 shows also the highest Cr%. This indicates that a close connection between AuNP formation and the iron and chromium release from the stainless steel exists. This is in agreement with the UV-VIS spectra reported in Figure 1b. In addition, the Cr/Fe and Ni/Fe ratios of the colloidal samples shown in Table 2 were more similar to the steel theoretical ones. In fact, Cr/Fe theoretical ratios are 0.2, 0.1 and 0.3 for AISI 430, 410, and 304 steels, respectively. Au4f region was also acquired on gold nanocolloids (Figure 3). Unlike previously reported spectra (Figure 2), three components were generally identified. The component around 84 eV is assigned to elemental gold, the ones around 85 eV and 87.5 eV are attributed to Au^(I)^ and Au^(III)^, respectively [48,49,50]. The component around 94.5 eV, indicated with an arrow in the graphs, is ascribed to Fe3s [48]. Au^(III)^ signal is ascribed to unreacted HAuCl_4_, in agreement with UV-Vis spectra registered on gold nanocolloids (Figure 1b). The presence of Au^(I)^ species can be explained in terms of uncomplete Au^(III)^ reduction, involving intermediate redox processes.

The relative abundance of gold species are reported in the Table 3.

Both 430- and 410-AuNPs showed the highest Au^(0)^ content, whereas Au^(I)^ was the most abundant species in 304-AuNPs. In all the samples, the presence of both Au^(I)^ and Au^(III)^ suggests that the reduction of Au^3+^ to Au^0^ is incomplete, mainly ascribable to the limited timescale of the synthesis.

In addition, the effect of aging on colloid stability was evaluated. 430-AuNPs and 410-AuNPs performed better than 304-AuNPs, whose precipitation occurred just after 24 h. 

**Figure 4:**
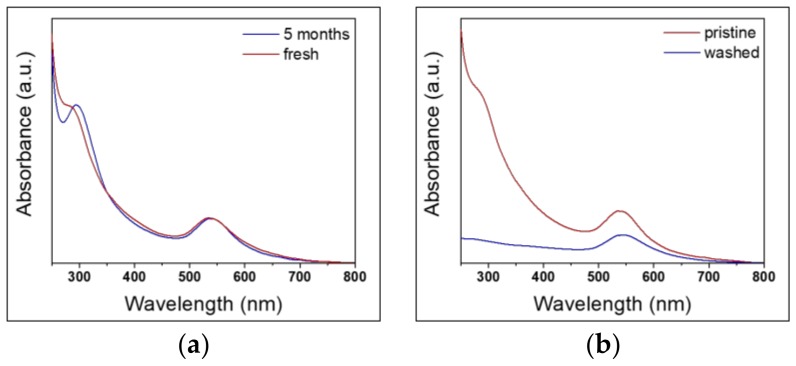
UV-Vis spectra of AuNPs. (**a**) 5-months aged 430-gold colloid compared with the fresh one; (**b**) washed 430-gold colloid compared with the pristine one.

As reported in Figure 4a, 430-AuNPs depict similar optical features after five months. Both UV-Vis spectra show the continuous absorption band in the range of 300−500 nm, ascribed to unreacted precursor and/or to hydroxides and chloride complexes of iron and chromium, as discussed earlier. These impurities contribute to AuNP stabilization. To investigate this, Au nanocolloids were centrifuged, washed three times, and re-dispersed in deionized water. Afterwards, UV-Vis spectra were registered.

**Figure 5:**
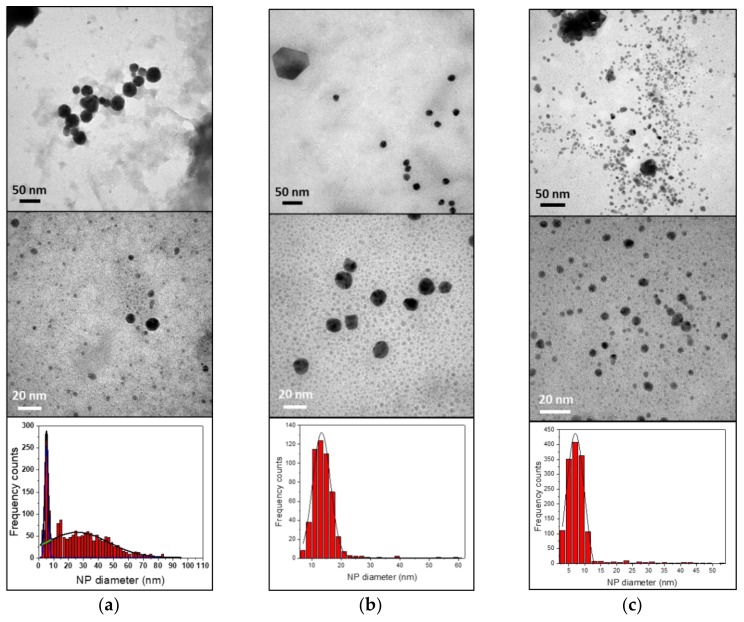
TEM images and corresponding size distribution histograms of AuNPs synthesized using different stainless steels: (**a**) AISI 430; (**b**) AISI 410; (**c**) AISI 304.

As reported in Figure 5b for the 430-AuNPs (taken as an example), a lower absorbance between 250−500 nm was visible, indicating that Fe/Cr complexes along with excess of gold precursor were removed. This finding was corroborated by XPS analysis performed on washed colloids (Table S3) as Fe% and Cr% reduced significantly. This result is in agreement with the evidences shown by López-Lorente and colleagues [51]. However, colloid washing resulted in AuNPs precipitation after two days. This fact confirms the key role played by the ions released from the stainless steel upon corrosion and formation of the AuNPs on the stabilization of the gold colloids. 

Au4f XPS high resolution region of the washed samples is reported in Figure S2, showing a single component centred at 83.8 ± 0.2 eV, ascribed to Au^(0)^. These evidences proved that the main impurities were washed away, together with the oxidized gold species.

TEM micrographs of all samples show spheroidal nanoparticles (Figure 5). 

Size distributions are relatively dissimilar, indicating different average size. Specifically, 430-AuNPs histogram was fitted with a bi-modal distribution, showing mean diameters of 5 ± 1 nm and 30 ± 20 nm. 410-AuNPs and 304-AuNPs had mean diameters of 13 ± 3 nm and 7 ± 3 nm, respectively. It is evident that the different steel composition affects NP morphology. 

Table 4 summarizes the typical features of the as-synthesized AuNPs.

The last column reports the ζ potential of gold colloids, which is typically positive, in agreement with previous work [30]. However, aforementioned work took into account only the AISI 304 steel and reported a higher value than our results due to different synthesis conditions (precursor concentration, temperature, reaction time). The low, slightly null value of ζ potential for 304-AuNPs is indicative of poor stability of such colloid that precipitates after two hours. In fact, it is reported that colloidal systems showing ζ potential above 25−30 mV (absolute value) are stable [52].

An estimation of the concentrations of the as-synthesized AuNPs (C_NP_) was done considering Lambert-Beer law (A = ε× b × C_NP_), using molar extinction coefficients ε reported in [53], as a function of NP size (as determined by TEM analysis), b = 1 cm (the optical path), and the absorbance A_LSPR_ at λ_LSPR_. The calculated concentration (C_NP_ = A_LSPR_/ε × b) of 430-AuNPs, 410-AuNPs and 304-AuNPs were about 2 nM, 0.6 nM and 0.3 nM, respectively, which are in the same concentration range of AuNPs synthesized by López-Lorente’s [31] research group. Those lower values compared to NP concentration obtained by Turkevich method [54,55,56] can be explained in terms of partial consumption of precursor in 15 min and reaction temperature. Increasing reaction times and/or temperature [31,57] can lead to an increment in the NP concentration.

On the basis of these results, the AISI 430 and 410 stainless steels seem to be the best candidate for the steel-assisted synthesis of AuNPs. In particular, they exhibit a lower corrosion resistance than AISI 304, and show good AuNP production yield, in combination with a better colloidal stability over time. Therefore, further investigations were carried out using the AISI 430 stainless steel. 

Considering the general advantages of potential-assisted electrochemical synthesis of nanoparticles [58], we also explored the option of applying an anodic potential to the stainless steel substrate. In this case, AuNPs synthesis was carried out applying an external potential to the stainless steel working electrode. The steel rod was put in a two-electrode system and a positive potential of 1 V was applied. The results showed that AuNPs LSPR bands had higher absorbance than those of colloids synthesized under typical conditions, suggesting an improved efficiency. Especially, the employment of an extra positive potential can overcome passivation and boost the corrosion of AISI 304 steel, ensuring a yield enhancement in the production of AuNPs (Supplementary Figure S3). Interestingly, the UV-vis spectra of those AuNPs prepared under the application of a potential of 1 V depicted an almost negligible band at around 300 nm, which may be related to quantitative reduction of Au(III) precursor.

### 3.2. Reaction Time

**Figure 6:**
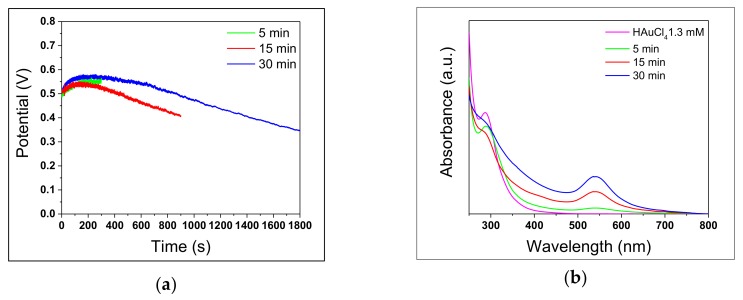
AuNPs synthesis performed with AISI 430 at different reaction times. (**a**) Open Circuit Potential (OCP) values, measured vs Ag/AgCl, KCl_sat_ reference electrode; (**b**) UV-Vis absorption spectra of the as-prepared colloids.

Steel-assisted synthesis of AuNPs was carried out with different reaction times, using AISI 430, to evaluate the progression of both steel corrosion and AuNP formation, as well as their influence on nanoparticle morphology. The evolution of OCP values was recorded to examine the electrochemical corrosion behaviour of AISI 430 in HAuCl_4_ solutions during the synthesis of AuNPs. OCP curves are reported in Figure 6a. In each case, they showed an initial increase of potential values, reaching a maximum at about 150 seconds, followed by a gradual decrease, suggesting the breakdown of natural passivation film and then a continuous corrosion progress over time. A comparison of the curves of Figure 6a relevant to the first five minutes of reaction provides an immediate visual estimation of the good process reproducibility. UV-Vis absorption spectra (Figure 6b) show the LSPR band ascribed to gold nanostructures, falling at 540 ± 2 nm [30,31]. The intensity of the LSPR band increases over the reaction time, whereas the precursor peak decreases. At the same time, however, the baseline in the range of 280−450 nm increases, indicating the progressive release of Fe^(III)^ and Cr^(III)^ species in solution.

**Figure 7:**
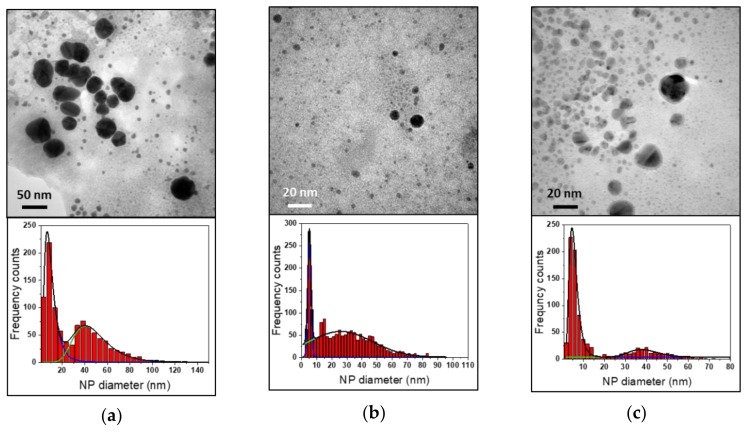
TEM images of AuNPs synthesized with AISI 430 at three different reaction times. (**a**) 5 minutes; (**b**) 15 minutes; (**c**) 30 minutes.

Figure 7 shows TEM images of gold colloids synthesized at different reaction times and their corresponding size distributions. 

In each case, AuNP were surrounded by a low-contrast thin layer; in particular, TEM images of AuNPs produced in 30 minutes showed a more evident thin film around NP agglomerates, compared with the other samples, due to the formation of hydroxides and chloride complexes of the iron and chromium released from the stainless steel. These findings agree with the increase with time of the UV-Vis band observed in the 280−400 nm range. As it can be seen, the NP size distributions were fitted with bi-modal distributions for all cases. Table 5 summarizes spectral and morphological details of synthesized AuNPs. 

Considering LSPR intensity observed after 5 minutes (Figure 6b, green line), it is clear that this reaction time is too short to obtain a satisfactory AuNP concentration. Absorbance increases significantly in the other two cases, indicating an increase in NP concentration. On the other hand, UV-Vis spectra in Figure 6b and TEM images in Figure 7 indicate that other species are formed, whose concentration increases with reaction time, causing an increment of impurities. However, LSPR positions were similar in all samples (correlating with similar size observed in the three samples). Finally, in each case the colloids presented a bi-modal size distribution. 

It was concluded that a short reaction time provides a scarce AuNPs yield. However, a reaction time of 30 minutes leads to the formation of an excessive amount of by-products. Therefore, a reaction time of 15 minutes was considered to be the best compromise to obtain a sufficiently high colloidal concentration with limited formation of iron and or chromium hydroxy-chloro-complexes.

### 3.3. Effect of Cl^−^ Concentration

**Figure 8:**
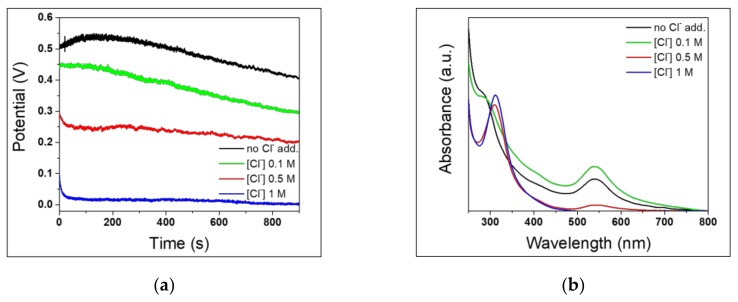
AuNPs synthesized with AISI 430 with different [Cl^−^]. (**a**) OCP measurements; (**b**) UV-Vis spectra.

According to Han et al. [30], Cl^−^ ions have a crucial role in the AuNP synthesis mechanism. The presence of Cl^−^ ions has surely a remarkable effect on the corrosion process of stainless steel [37]. It is well known that Cl^−^ ions can induce the formation of pits on oxide film on stainless steel surface, ensuring passivation film break and promoting the corrosion mechanism and the dissolution of metal ions in the solution [59,60,61]. In theory, this reflects a decreasing in pitting potential values (E_pit_) which delimits the trans-passivation region of Evans diagram [62]. The critical potential E_pit_ corresponds to the Cl^−^ concentration needed to displace adsorbed oxygen species on steel surface and facilitate the oxidation of iron atoms [38]. Since steel-assisted synthesis of AuNPs ought to rely on steel corrosion efficiency, so also on pitting site concentration, we followed the OCP during AuNP syntheses, which were carried out using three different chloride ion concentrations. We observed that the initial OCP values decrease as Cl^−^ concentration increases (Figure 8a). The use of different Cl^−^ concentrations also affects the OCP changes on reaction time.

**Figure 9:**
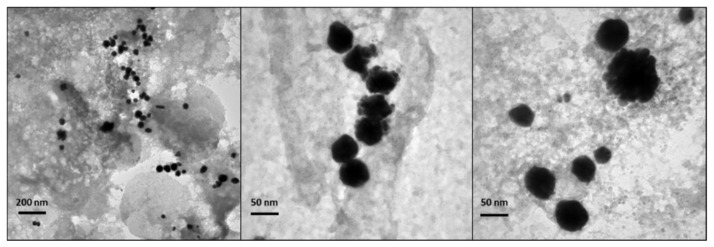
TEM images of 430-AuNPs synthesized with NaCl 0.1 M.

At the highest Cl^−^ concentrations of 1 M and 0.5 M, a constant potential value seemed to be immediately reached in the first minutes, unlike with 0.1 M NaCl solution, in which it gradually decreased over 15 minutes. Due to their small radius and the strong adsorbing and penetrating power, Cl^−^ ions can damage the passivation film and promote local corrosion [39]. For this reason, a higher Cl^−^ concentration allows eroding the oxide film more rapidly, easily obtaining more negative and constant corrosion values of potential. This can be considered beneficial from the corrosion point of view, e.g. for promoting the reaction of the stainless-steel substrate. In fact, the higher the [Cl^−^], the lower the OCP suggesting promoted corrosion. Interestingly, increasing chloride concentration did not significantly improve AuNP production. This is clear from the UV-Vis spectra in Figure 8b. In fact, at chloride concentration ≥ 0.5 M, gold LSPR signal was significantly suppressed, due also to the poor colloidal stability at high ionic strengths. The addition of NaCl 0.1 M had a limited positive influence on nanoparticle synthesis. TEM analysis of 430-AuNPs prepared in the presence of NaCl 0.1 M is shown in Figure 9. 

In this case, nanoparticles are surrounded by a very high amount of low-contrast phase, attributed to metal hydroxy-chloro-complexes.

The use of NaCl 0.1 M was considered beneficial only in the case of syntheses carried out with AISI 304. In fact, in the case of AISI 410, no notable improvements were observed. On the other hand, LSPR absorbance increase for 304-AuNPs was remarkable (Supplementary Figure S4), with Cl^−^ addition contributing to reduce dramatically the AISI 304 resistance to corrosion.

### 3.4. Effect of pH

**Figure 10:**
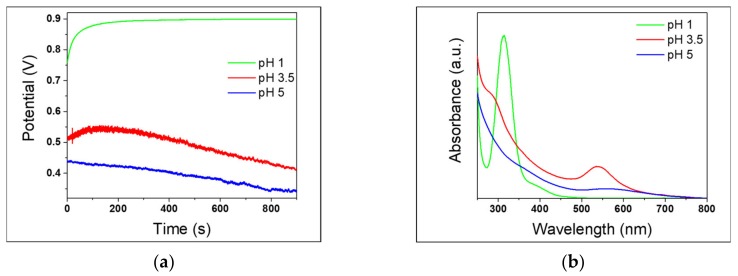
430-AuNPs synthesis carried out at different pH. (**a**) OCP measurements; (**b**) UV-Vis spectra.

In order to investigate the possible role of H^+^ ions in the production mechanism [31], syntheses at different pH values were carried out using AISI 430 samples, recording the OCP curves under *operando* conditions. All previous AuNPs syntheses were carried out at an acidic pH of about 3.5, which remained constant over synthesis time, in agreement with previous works [31]. In this sense, hydrogen works as electron mediator between steel elements and AuCl_4_^-^. Hydrogen is formed as a consequence of the reduction of protons of the acidic media mediated by the steel substrate; then it reduces AuCl_4_^-^ ions yielding AuNPs [31]. In order to investigate a wider acidic pH range, steel-assisted syntheses of AuNPs were performed at pH values of about 1 and 5. Figure 10a shows the relevant OCP measurement. Data suggest that the corrosion mechanism was promoted by weakly acidic pH values. However, it is known that when the pH increases towards less acidic values the equilibrium of gold complexes, arising from chloroauric acid, shifts toward more hydrolysed forms [63,64,65]. This restricts the presence of AuCl_4_¯ species, whose reduction heads the seed particle formation. As a matter of fact, the LSPR absorbance of gold colloids produced at pH around 5 is lower than the one obtained at pH 3.5 (Figure 10b). On the other hand, at extremely low pH values, the potential values were more positive, and the UV-Vis spectrum did not show any significant AuNP LSPR band. This result could be due to the higher stability of AuCl_4_¯ complexes at low pH [63], which slows down or inhibits the reduction to Au(0), limiting seed particle formation [64]. 

### 3.5. Decoupling the Effects related to the Electrode and the Solution in the OCP Measurements

**Figure 11:**
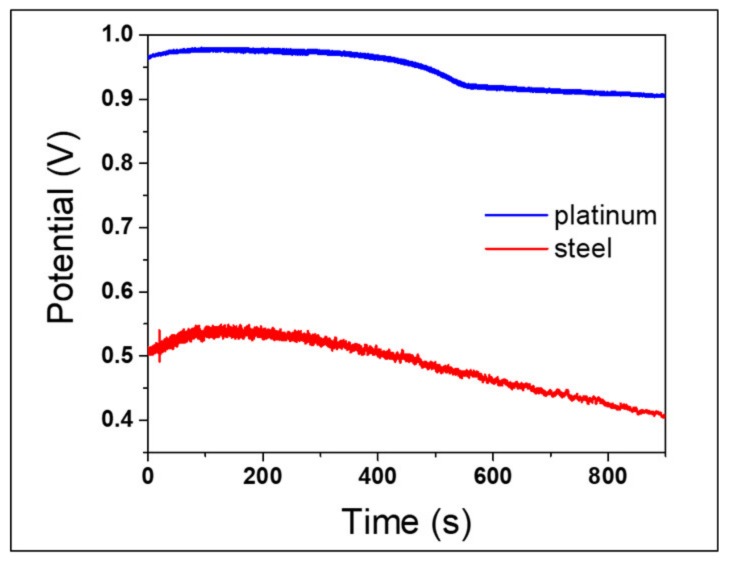
OCP measurements recorded using platinum wire (blue) and steel wire (red) as working electrode.

The recorded potentials during OCP measurements are a combination of reduction potential of AuCl_4_¯ ions in solution and corrosion potential of stainless steel, being observed using a steel wire as working electrode. Therefore, we carried out OCP measurements using a platinum wire as a redox-inert working electrode, in order to outline what could be ascribable exclusively to AuCl_4_¯ reduction. The resulting OCP curve is shown in Figure 11, where it is compared with the homologous curve, obtained in the presence of the stainless-steel electrode.

The blue curve, representing solution potential, reported initial potential value just below 1 V. Instead, the steel OCP (red) curve starts with a potential value around 0.5 V, which is the superimposition of solution and steel corrosion potentials. In a very preliminary first approximation, subtracting steel potential values to platinum ones, we can roughly identify potential values exclusively relevant to steel corrosion, which are about −0.5 V, and are rather stationary over reaction time. This is in agreement with typical AISI 430 corrosion potential established by Evans diagram [66,67]. So, the decrement that is clearly observable in OCP measurement of steel used for AuNPs synthesis should be strictly due to the variation of solution potential, caused by the progress of the AuCl_4_¯ reduction reaction. 

According to the overall experimental work and the relevant results, it can be highlighted that the proposed procedure for AuNP production suits very well when AISI 430 is used as reducing component in combination with a diluted Au(III) salt solution as precursor. OCP measurements allowed investigating the role of steel composition as well as of corrosion on NP synthesis. It seems that neither the external potential nor chloride addition contribute significantly to the process, when a sufficiently corrodible steel is used. AuNPs of size around 30 nm with positive surface potential are formed with mainly spheroidal morphology. A reaction time of 15 minutes gives reliable results in terms of AuNP purity and yield. XPS data indicate that Au content correlates strongly with iron content more than with chromium amount. Such a result agrees with the formation of iron and chromium hydroxy-chloro-complexes, as suggested by the increase of the absorption band in the 280–400 nm range observed by UV-Vis spectroscopic characterization. Such species are also responsible for the positive zeta-potential values as well as for NP stability up to 5 months. Au(0) represents the main chemical environment in 430-AuNPs, though additional species are still present in solution. Their removal can be performed by centrifugation, although this reduces their stability.

## 4. Conclusions

Gold nanoparticles were synthesized using a simple, one-pot, low cost, and scalable method, based on the reduction of Au^3+^ → Au^0^ from a stainless steel rod. In order to contribute to the investigation of this system, we performed the AuNP syntheses monitoring the overall open circuit potential (OCP), to explore the electrochemical behaviour of steel and track the solution potential. Different experimental conditions were also examined. Specifically, the syntheses of AuNPs using stainless steel with different compositions were investigated and we found that the 410-AuNPs showed features comparable with 430-AuNPs. UV-vis spectra revealed the formation of the AuNPs along with the release of iron and chromium species from stainless steel, as confirmed by XPS. On the other hand, synthesis assisted by AISI 304 steel produced the lowest AuNP concentration, due to its better corrosion resistance. In the latter case, we observed that the addition of NaCl 0.1 M seems to improve the synthesis yield, due to the Cl^−^ capability to pit the steel surface. Hence, the results highlight the crucial role of corrosion mechanism in the AuNP formation because a better steel surface corrosion provides a higher AuNP concentration. The easier tendency to corrosion of 430 steel than 304 allows to produce a higher concentration of AuNPs. Furthermore, the addition of an element that assists the steel surface pitting, leads to an increase in the corrosion inclination of steel, providing enhanced AuNP production. On the other hand, the effect of the pH of the precursor solution affects the equilibrium of gold complex, arising from chloroauric acid, toward more hydrolysed forms, restricting the presence of AuCl_4_¯ complexes, whose reduction heads the seed particle formation. In conclusion, the investigation of gold colloid production mechanism was explored and the influence of some experimental parameters was tested, demonstrating the capability to synthesized AuNPs with several features. The proposed method allows for the production of stable AuNPs in a simple, fast, and easy way, especially using AISI 430 or AISI 410 as solid reductant. During the AuNP synthesis, some by-products, namely Fe^3+^ and Cr^3+^ hydroxides and chloride mixed complexes, are formed. Such finding was corroborated by UV-Vis, TEM and XPS analyses. However, few washing steps can lead to the elimination of such species. As a result, purified AuNPs can be used in real applications. Preliminary studies were performed to investigate the modification of silicon nanowires with as-synthesized AuNPs for their potential application in sensor development.

## Figures and Tables

**Table 1:** Surface chemical composition of steel surfaces before and after their use, obtained by XPS. The main elements are reported; the complete dataset is shown in Table S1. Error is expressed as the larger value between the error associated to a single quantification and one standard deviation; error on Fe, Si, Cr, Ni and Au percentages is ± 0.2%; error on the abundance of other elements is ± 0.5%. Cr/Fe, Au/Fe and Ni/Fe ratios of the same samples are showed, as well.

	430		410		304	
	Before Use	After Use	Before Use	After Use	Before Use	After Use
**Au%**	/	17.4	/	30.1	/	5.5
**Fe%**	1.2	8.2	2.9	6	2.9	5.2
**Cr%**	1.3	4.2	1.1	2.4	1.3	2.2
**Ni%**	/	/	/	/	0.3	< 0.2
**Cr/Fe**	1.1 ± 0.3	0.5 ± 0.1	0.4 ± 0.1	0.4 ± 0.1	0.4 ± 0.1	0.4 ± 0.1
**Au/Fe**		2.1 ± 0.1		5.0 ± 0.2		1.1 ± 0.1
**Ni/Fe**					~ 0	~ 0

**Table 2:** Surface chemical composition of colloids synthesized with AISI 430, 410 and 304, obtained by XPS. The main elements are reported; the complete dataset is shown in Table S2. Error is expressed as the larger value between the error associated to a single quantification and one standard deviation; error on Fe, Si, Cr, Ni and Au percentages is ± 0.2%; error on the abundance of other elements is ± 0.5%. Cr/Fe, Au/Fe and Ni/Fe ratios of the same samples are showed, as well.

	430-AuNPs	410-AuNPs	304-AuNPs
**Au%**	1.4	0.6	1.0
**Fe%**	7.2	3.8	4.1
**Cr%**	1.8	1.1	1.0
**Ni%**			0.3
**Cr/Fe**	0.3 ± 0.1	0.3 ± 0.2	0.2 ± 0.1
**Au/Fe**	0.2 ± 0.1	0.2 ± 0.1	0.2 ± 0.1
**Ni/Fe**			~ 0

**Table 3:** Relative abundance of gold chemical states relevant to the gold colloids synthesized with AISI 430, 410 and 304.

	rel. %		
Sample	Au^(0)^	Au^(I)^	Au^(III)^
**430-AuNPs**	39.5	29.5	31.0
**410-AuNPs**	48.0	25.6	26.4
**304-AuNPs**	37.9	43.8	18.3

**Table 4:** LSPR position (λ_SPR_), absorbance (Abs.) at λ_LSPR_, AuNP mean diameters determined by TEM analysis (d_TEM_), and zeta potential values (ζ potential) obtained for 430-AuNPs, 410-AuNPs, and 304-AuNPs colloids.

Sample ID	λ_LSPR_ (nm)	Abs. (a.u.)	d_TEM_ (nm)	ζ Potential (mV)
**430-AuNPs**	538 ± 3	0.690 ± 0.004	5 ± 1 30 ± 20	38.7 ± 0.7
**410-AuNPs**	544 ± 4	1.004 ± 0.004	13 ± 3	40.6 ± 3.7
**304-AuNPs**	538 ± 4	0.115 ± 0.004	7 ± 3	1.9 ± 0.7

**Table 5:** UV-Vis ad TEM details of AuNPs.

Reaction Time	LSPR Position (nm)	Abs. (a.u.)	d_TEM_ (nm)	
**5’**	541 ± 2	0.100 ± 0.004	9 ± 6	46 ± 24
**15’**	538 ± 3	0.500 ± 0.004	5 ± 1	26 ± 21
**30’**	541 ± 2	0.700 ± 0.004	5 ± 3	39 ± 20

## Supplementary Materials

The following are available online at https://www.mdpi.com/2079-4991/10/4/622/s1, Figure S1: Experimental setup for AuNPs syntheses; Figure S2: Au4f high resolution regions in washed 430-AuNP colloid, Figure S3: UV-Vis absorption spectra of AuNPs synthetized applied an external potential to stainless steel, Figure S4: Comparison of UV-Vis absorption spectra of gold colloids synthetized using AISI 304 stainless steel, with and without adding NaCl, Table S1: Surface chemical composition of steel surface before and after their use, obtained by XPS, Table S2: Surface chemical composition of colloids synthesized with AISI 430, 410 and 304, obtained by XPS, Table S3: Surface chemical composition of washed gold nanocolloids, obtained by XPS.

## References

[1] Amendola V., Pilot R., Frasconi M., Maragò O.M., Iatì M.A. (2017). Surface plasmon resonance in gold nanoparticles: a review. J. Phys. Condens. Matter.

[2] Saha K., Agasti S.S., Kim C., Li X., Rotello V.M. (2012). Gold Nanoparticles in Chemical and Biological Sensing. Chem. Rev..

[3] Hu M., Chen J., Li Z.-Y., Au L., Hartland G.V., Li X., Marquez M., Xia Y. (2006). Gold nanostructures: engineering their plasmonic properties for biomedical applications. Chem. Soc. Rev..

[4] Boisselier E., Astruc D. (2009). Gold nanoparticles in nanomedicine: preparations, imaging, diagnostics, therapies and toxicity. Chem. Soc. Rev..

[5] Dreaden E.C., Alkilany A.M., Huang X., Murphy C.J., El-Sayed M.A. (2012). The golden age: Gold nanoparticles for biomedicine. Chem. Soc. Rev..

[6] Daniel M.-C., Astruc D. (2004). Gold Nanoparticles: Assembly, Supramolecular Chemistry, Quantum-Size-Related Properties, and Applications toward Biology, Catalysis, and Nanotechnology. Chem. Rev..

[7] Johannsmeier S., Heeger P., Terakawa M., Kalies S., Heisterkamp A., Ripken T., Heinemann D. (2018). Gold nanoparticle-mediated laser stimulation induces a complex stress response in neuronal cells. Sci. Rep..

[8] Riley R.S., Day E.S. (2017). Gold nanoparticle-mediated photothermal therapy: applications and opportunities for multimodal cancer treatment. Wiley Interdiscip. Rev. Nanomed. Nanobiotechnol..

[9] Baek S., Singh R.K., Kim T.-H., Seo J., Shin U.S., Chrzanowski W., Kim H.-W. (2016). Triple Hit with Drug Carriers: pH- and Temperature-Responsive Theranostics for Multimodal Chemo- and Photothermal Therapy and Diagnostic Applications. ACS Appl. Mater. Interfaces.

[10] Kim T., Lee N., Arifin D.R., Shats I., Janowski M., Walczak P., Hyeon T., Bulte J.W.M. (2017). In Vivo Micro-CT Imaging of Human Mesenchymal Stem Cells Labeled with Gold-Poly-l-Lysine Nanocomplexes. Adv. Funct. Mater..

[11] Manivasagan P., Bharathiraja S., Bui N.Q., Jang B., Oh Y.-O., Lim I.G., Oh J. (2016). Doxorubicin-loaded fucoidan capped gold nanoparticles for drug delivery and photoacoustic imaging. Int. J. Biol. Macromol..

[12] Chen Y., Xianyu Y., Jiang X. (2017). Surface Modification of Gold Nanoparticles with Small Molecules for Biochemical Analysis. Acc. Chem. Res..

[13] Khalil I., Julkapli N.M., Yehye W.A., Basirun W.J., Bhargava S.K. (2016). Graphene–Gold Nanoparticles Hybrid—Synthesis, Functionalization, and Application in a Electrochemical and Surface-Enhanced Raman Scattering Biosensor. Materials.

[14] Liu P., Han L., Wang F., Petrenko V.A., Liu A. (2016). Gold nanoprobe functionalized with specific fusion protein selection from phage display and its application in rapid, selective and sensitive colorimetric biosensing of Staphylococcus aureus. Biosens. Bioelectron..

[15] López-Lorente Á.I., Izquierdo J., Kranz C., Mizaikoff B. (2017). Boron-doped diamond modified with gold nanoparticles for the characterization of bovine serum albumin protein. Vib. Spectrosc..

[16] López-Lorente Á.I., Wang P., Mizaikoff B. (2017). Towards label-free mid-infrared protein assays: in-situ formation of bare gold nanoparticles for surface enhanced infrared absorption spectroscopy of bovine serum albumin. Microchim Acta.

[17] Turkevich J., Stevenson P.C., Hillier J. (1951). A study of the nucleation and growth processes in the synthesis of colloidal gold. Discuss. Faraday Soc..

[18] Brust M., Walker M., Bethell D., Schiffrin D.J., Whyman R. (1994). Synthesis of thiol-derivatised gold nanoparticles in a two-phase Liquid–Liquid system. J. Chem. Soc. Chem. Commun..

[19] Tao C. (2018). Antimicrobial activity and toxicity of gold nanoparticles: research progress, challenges and prospects. Lett. Appl. Microbiol..

[20] Sengani M., Grumezescu A.M., Rajeswari V.D. (2017). Recent trends and methodologies in gold nanoparticle synthesis—A prospective review on drug delivery aspect. OpenNano.

[21] Freitas de Freitas L., Varca G.H.C., Dos Santos Batista J.G., Benévolo Lugão A. (2018). An Overview of the Synthesis of Gold Nanoparticles Using Radiation Technologies. Nanomaterials.

[22] Zhao P., Li N., Astruc D. (2013). State of the art in gold nanoparticle synthesis. Coord. Chem. Rev..

[23] Sportelli M.C., Izzi M., Volpe A., Clemente M., Picca R.A., Ancona A., Lugarà P.M., Palazzo G., Cioffi N. (2018). The Pros and Cons of the Use of Laser Ablation Synthesis for the Production of Silver Nano-Antimicrobials. Antibiotics.

[24] Izzi M., Sportelli M.C., Ditaranto N., Picca R.A., Innocenti M., Sabbatini L., Cioffi N. (2020). Pros and Cons of Sacrificial Anode Electrolysis for the Preparation of Transition Metal Colloids: A Review. ChemElectroChem.

[25] Cioffi N., Colaianni L., Ieva E., Pilolli R., Ditaranto N., Angione M.D., Cotrone S., Buchholt K., Spetz A.L., Sabbatini L. (2011). Electrosynthesis and characterization of gold nanoparticles for electronic capacitance sensing of pollutants. Electrochim. Acta.

[26] Shedbalkar U., Singh R., Wadhwani S., Gaidhani S., Chopade B.A. (2014). Microbial synthesis of gold nanoparticles: Current status and future prospects. Adv. Colloid Interface Sci..

[27] Singh P., Kim Y.-J., Zhang D., Yang D.-C. (2016). Biological Synthesis of Nanoparticles from Plants and Microorganisms. Trends Biotechnol..

[28] Patra S., Madhuri R., Kanchi S., Ahmed S. (2018). Green Synthesis of Noble Metal Nanoparticles: A Step Forward to Economical and Sustainable Development. Green Metal Nanoparticles: Synthesis, Characterization and Their aApplications.

[29] Das R.K., Pachapur V.L., Lonappan L., Naghdi M., Pulicharla R., Maiti S., Cledon M., Dalila L.M.A., Sarma S.J., Brar S.K. (2017). Biological synthesis of metallic nanoparticles: plants, animals and microbial aspects. Nanotechnol. Environ. Eng..

[30] Han T.H., Khan M.M., Kalathil S., Lee J., Cho M.H. (2013). Synthesis of Positively Charged Gold Nanoparticles Using a Stainless-Steel Mesh. J. Nanosci. Nanotechnol..

[31] López-Lorente A.I., Simonet B.M., Valcárcel M., Eppler S., Schindl R., Kranz C., Mizaikoff B. (2014). Characterization of stainless steel assisted bare gold nanoparticles and their analytical potential. Talanta.

[32] Han T.H., Khan M.M., Lee J., Cho M.H. (2014). Optimization of positively charged gold nanoparticles synthesized using a stainless-steel mesh and its application for colorimetric hydrogen peroxide detection. J. Ind. Eng. Chem..

[33] ImageJ. https://imagej.nih.gov/ij/.

[34] Casiello M., Picca R.A., Fusco C., D’Accolti L., Leonardi A.A., Lo Faro M.J., Irrera A., Trusso S., Cotugno P., Sportelli M.C. (2018). Catalytic Activity of Silicon Nanowires Decorated with Gold and Copper Nanoparticles Deposited by Pulsed Laser Ablation. Nanomaterials.

[35] Picca R.A., Calvano C.D., Faro M.J.L., Fazio B., Trusso S., Ossi P.M., Neri F., D’Andrea C., Irrera A., Cioffi N. (2016). Functionalization of silicon nanowire arrays by silver nanoparticles for the laser desorption ionization mass spectrometry analysis of vegetable oils. J. Mass Spectrom..

[36] McCafferty E., McCafferty E. (2010). Thermodynamics of Corrosion: Electrochemical Cells and Galvanic Corrosion. Introduction to Corrosion Science.

[37] Malik A.U., Mayan Kutty P.C., Siddiqi N.A., Andijani I.N., Ahmed S. (1992). The influence of pH and chloride concentration on the corrosion behaviour of AISI 316L steel in aqueous solutions. Corros. Sci..

[38] Loto R.T., Joseph O.O., Akanji O. (2015). Electrochemical corrosion behaviour of austenitic stainless steel (type 304) in dilute hydrochloric acid solution. J. Mater. Environ. Sci..

[39] Liu B.Y., Xue Y.J., Yang Z.H., Fang X.X. (2016). Corrosion Behavior of Austenitic Stainless Steel in Hydrochloric Acid Solution and Flow. Mater. Sci. Forum.

[40] Jeon S.-H., Kim S.-T., Lee J.-S., Lee I.-S., Park Y.-S. (2012). Effects of Sulfur Addition on the Formation of Inclusions and the Corrosion Behavior of Super Duplex Stainless Steels in Chloride Solutions of Different pH. Mater. Trans..

[41] Gangopadhayay A.K., Chakravorty A. (1961). Charge Transfer Spectra of some Gold(III) Complexes. J. Chem. Phys..

[42] Gamlen G.A., Jordan D.O. (1953). 295. A spectrophotometric study of the iron(III) chloro-complexes. J. Chem. Soc..

[43] Loures C.C.A., Alcântara M.A.K., Filho H.J.I., Teixeira A.C.S.C., Silva F.T., Paiva T.C.B., Samanamud G.R.L. (2013). Advanced Oxidative Degradation Processes: Fundamentals and Applications. Int. Rev. of Chem. Eng. (IRECHE).

[44] Muramatsu A., Kanie K., Waseda Y., Suzuki S. (2006). Mechanistic Study on Formation of Iron Hydroxides and Oxides with FT-IR and UV Photospectroscopy. Characterization of Corrosion Products on Steel Surfaces.

[45] Mischler S., Vogel A., Mathieu H.J., Landolt D. (1991). The chemical composition of the passive film on Fe-24Cr and Fe-24Cr-11Mo studied by AES, XPS and SIMS. Corros. Sci..

[46] Lorang G., Belo M.D.C., Simões A.M.P., Ferreira M.G.S. (1994). Chemical Composition of Passive Films on AISI 304 Stainless Steel. J. Electrochem. Soc..

[47] Suzuki S., Nakazawa T., Waseda Y. (1996). Chromium and Nitrogen Segregation in Thin Oxide Layers Formed on the Surface of 17Cr-Ni-Mo-N Austenitic Steels Studied by Angle Resolved XPS. ISIJ Int..

[48] NIST X-ray Photoelectron Spectroscopy Database, Version 4.1 (National Institute of Standards and Technology, Gaithersburg, 2012). http://srdata.nist.gov/xps/.

[49] Clukay C.J., Grabill C.N., Hettinger M.A., Dutta A., Freppon D.J., Robledo A., Heinrich H., Bhattacharya A., Kuebler S.M. (2014). Controlling formation of gold nanoparticles generated in situ at a polymeric surface. Appl. Surf. Sci..

[50] Kitagawa H., Kojima N., Nakajima T. (1991). Studies of mixed-valence states in three-dimensional halogen-bridged gold compounds, Cs2AuIAuIIIX6, (X = Cl, Br or I). Part 2. X-Ray photoelectron spectroscopic study. J. Chem. Soc. Dalton Trans..

[51] López-Lorente Á.I., Cárdenas S., González-Sánchez Z.I. (2019). Effect of synthesis, purification and growth determination methods on the antibacterial and antifungal activity of gold nanoparticles. Mater. Sci. Eng., C.

[52] Kumar A., Dixit C.K., Nimesh S., Chandra R., Gupta N. (2017). 3 - Methods for characterization of nanoparticles. Advances in Nanomedicine for the Delivery of Therapeutic Nucleic Acids.

[53] Tang J., Gao K., Ou Q., Fu X., Man S.-Q., Guo J., Liu Y. (2018). Calculation extinction cross sections and molar attenuation coefficient of small gold nanoparticles and experimental observation of their UV–vis spectral properties. Spectrochim. Acta Part A Mol. Biomol. Spectrosc..

[54] Roca M., Haes A.J. (2008). Silica−Void−Gold Nanoparticles: Temporally Stable Surface-Enhanced Raman Scattering Substrates. J. Am. Chem. Soc..

[55] Huang D., Niu C., Wang X., Lv X., Zeng G. (2013). “Turn-On” Fluorescent Sensor for Hg^2+^ Based on Single-Stranded DNA Functionalized Mn:CdS/ZnS Quantum Dots and Gold Nanoparticles by Time-Gated Mode. Anal. Chem..

[56] Zakaria H.M., Shah A., Konieczny M., Hoffmann J.A., Nijdam A.J., Reeves M.E. (2013). Small Molecule- and Amino Acid-Induced Aggregation of Gold Nanoparticles. Langmuir.

[57] López-Lorente Á.I., Valcárcel M., Mizaikoff B. (2014). Continuous flow synthesis and characterization of tailor-made bare gold nanoparticles for use in SERS. Microchim. Acta.

[58] Reetz M.T., Helbig W. (1994). Size-Selective Synthesis of Nanostructured Transition Metal Clusters. J. Am. Chem. Soc..

[59] Hoar T.P., Jacob W.R. (1967). Breakdown of Passivity of Stainless Steel by Halide Ions. Nature.

[60] Burstein G.T., Pistorius P.C., Mattin S.P. (1993). The nucleation and growth of corrosion pits on stainless steel. Corros. Sci..

[61] Wilde B.E., Williams E. (1971). The use of current/voltage curves for the study of localized corrosion and passivity breakdown on stainless steels in chloride media. Electrochim. Acta.

[62] Landolt D. (2007). Localized Corrosion Phenomena. Corrosion and Surface Chemistry of Metals.

[63] Pacławski K., Sak T. Kinetics and Mechanism of the Reaction of Gold(III) Chloride Complexes with Formic Acid. https://www.ingentaconnect.com/content/doaj/14505339/2015/00000051/00000002/art00004.

[64] Wojnicki M., Rudnik E., Luty-Błocho M., Pacławski K., Fitzner K. (2012). Kinetic studies of gold(III) chloride complex reduction and solid phase precipitation in acidic aqueous system using dimethylamine borane as reducing agent. Hydrometallurgy.

[65] Wuithschick M., Birnbaum A., Witte S., Sztucki M., Vainio U., Pinna N., Rademann K., Emmerling F., Kraehnert R., Polte J. (2015). Turkevich in New Robes: Key Questions Answered for the Most Common Gold Nanoparticle Synthesis. ACS Nano.

[66] McCafferty E., McCafferty E. (2010). Passivity. Introduction to Corrosion Science.

[67] Biefer G.J. (1970). Effects of alloying on polarization and corrosion of Type 430 stainless steel. Can. Metall. Q..

