# Supplementary material for: Gold Nanoparticles Synthesis Using Stainless Steel as Solid Reductant: A Critical Overview
Source: Nanomaterials (Basel). 2020 Mar 27;10(4):622. doi: 10.3390/nano10040622 (PMC7221709; doi:10.3390/nano10040622)
Supplement: Supplementary file 1 [file nanomaterials-10-00622-s001.pdf]

Supporting Info

Gold Nanoparticles Synthesis Using Stainless Steel as Solid Reductant: A Critical Overview

Margherita Izzi [1], Maria C. Sportelli [1], Luciana Tursellino [1], Gerardo Palazzo [1], Rosaria A. Picca [1,*], Nicola Cioffi [1,*] and Ángela I. López Lorente [2]

[1] Department of Chemistry, University of Bari "Aldo Moro", Via Orabona, 4, 70126 Bari, Italy; margherita.izzi@uniba.it (M.I.), maria.sportelli@uniba.it (M.C.S.), lucianakturs@gmail.com (L.T.), gerardo.palazzo@uniba.it (G.P.)

[2] Departamento de Química Analítica, Instituto Universitario de Investigación en Química Fina y Nanoquímica IUIQFN, Universidad de Córdoba, Campus de Rabanales, Edificio Marie Curie, E-14071 Córdoba, Spain; q32loloa@uco.es

* Correspondence: rosaria.picca@uniba.it (R.A.P.); nicola.cioffi@uniba.it (N.C.)

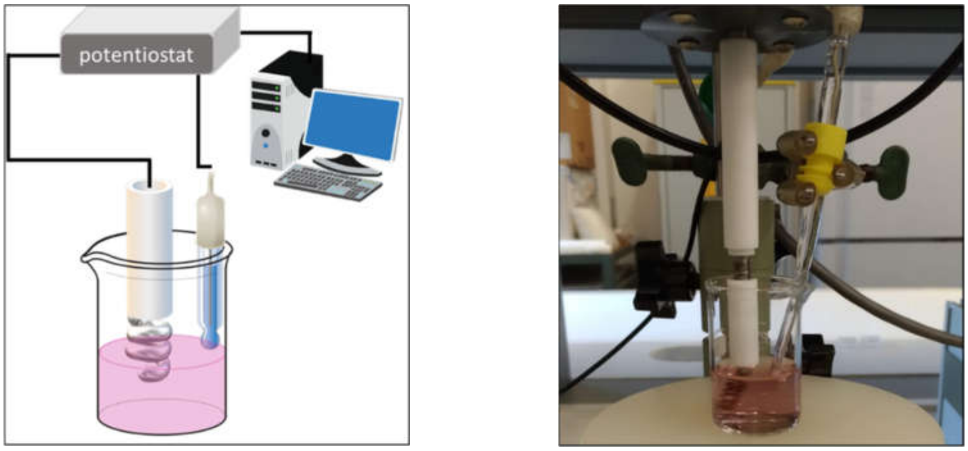

(**a**) (**b**)

Figure 1. Experimental setup for AuNPs syntheses. (**a**) Schematic representation; (**b**) real setup.

Table S1. Surface chemical composition of steel surface before and after their use, obtained by XPS. Error is expressed as the larger value between the error associated to a single quantification and one standard deviation; error on Fe, Si, Cr, Ni, and Au percentages is ± 0.2%; error on the abundance of other elements is ± 0.5%.

	430		410		304	
	Not Used	Used	Not Used	Used	Not Used	Used
C%	58.1	41.4	59.5	37.1	67.1	55.8
O%	28.5	26.0	29.6	23.0	23.0	27.2
Si%	9.0	/	2.9	3.7	1.4	2.4
Au%	/	17.4	/	30.1	/	5.5
Fe%	1.2	8.2	2.9	6	2.9	5.2
Cr%	1.3	4.2	1.1	2.4	1.3	2.2
Cl%	0.4	2.9	0.5	1.5	1.3	1.5
Na%	0.3	/	2.1	0.5	/	/
Ca%	1.2	/	1.4	/	2.2	/
Ni%	/	/	/	/	0.3	< 0.2

Table S2. Surface chemical composition of colloids synthesized with AISI 430, 410, and 304, obtained by XPS. Error is expressed as the larger value between the error associated to a single quantification and one standard deviation; error on Fe, Si, Cr, Ni, and Au percentages is ± 0.2%; error on the abundance of other elements is ± 0.5%.

	430-AuNPs	410-AuNPs	304-AuNPs
**C%**	33.5	29.4	39.8
**O%**	34.7	33.8	32.5
**Si%**	7.9	18.3	18.3
**Au%**	1.4	0.6	1.0
**Fe%**	7.2	3.8	4.1
**Cr%**	1.8	1.1	1.0
**Cl%**	10.4	8.5	5.3
**Na%**	0.4	0.6	
**N%**	0.9	1.0	
**Mg%**	1.8	3.2	1.8
**Ni%**			0.3

Table S3. Surface chemical composition of washed gold nanocolloids, obtained by XPS. Error is expressed as the larger value between the error associated to a single quantification and one standard deviation; error on Fe, Si, Cr, and Au percentages is ± 0.2%; error on the abundance of other elements is ± 0.5%

	**At. %**
	**washed**
**C**	22.6
**O**	37.6
**Si**	34.8
**Au**	1.0
**Fe**	1.6
**Cr**	0.3
**Cl**	0.8

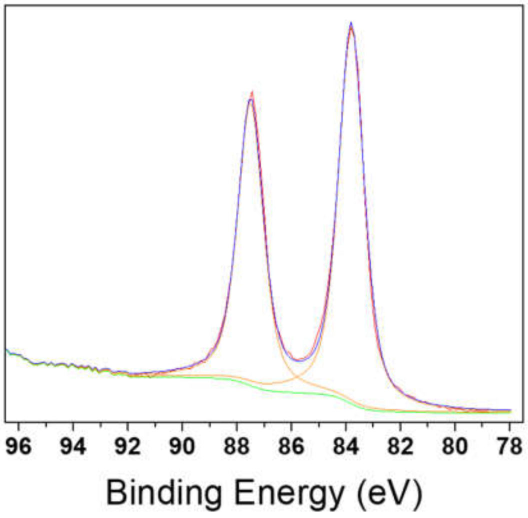

Figure S2. Au4f high resolution regions in washed 430-AuNP colloid.

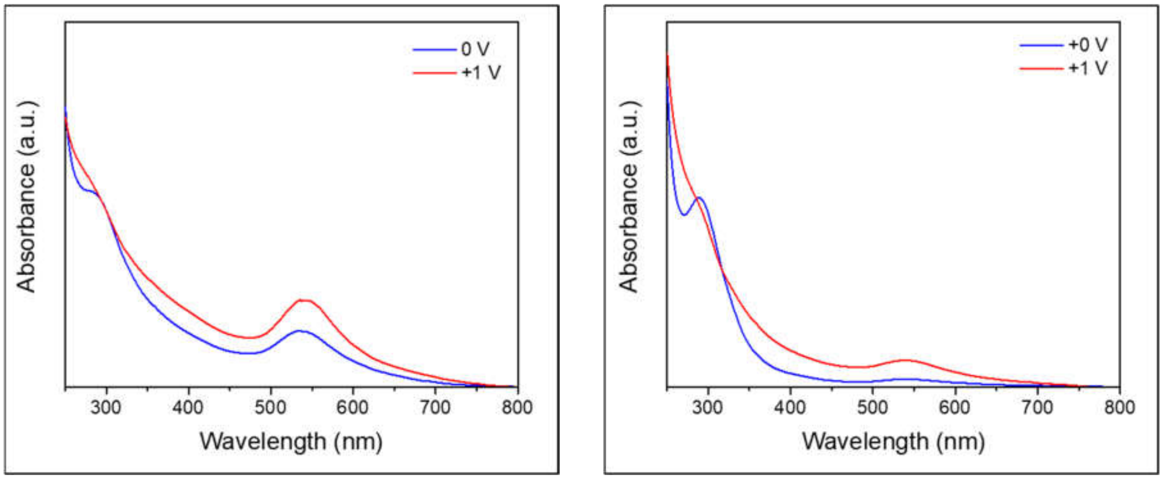

(**a**) (**b**)

Figure S3. UV-Vis absorption spectra of AuNPs synthetized applied an external potential to stainless steel: (**a**) AISI 430; (**b**) AISI 304.

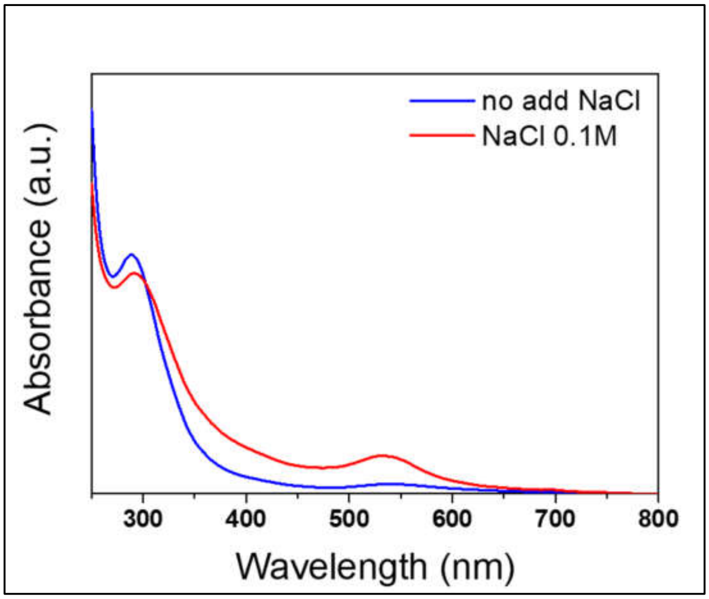

Figure S4. Comparison of UV-Vis absorption spectra of gold colloids synthetized using AISI 304 stainless steel, with and without adding NaCl.
